# A study on the effect of radiographic angles on clubfoot’s recurrence

**DOI:** 10.1038/s41598-023-38882-4

**Published:** 2023-07-20

**Authors:** Mohammad Ali Tahririan, Sara Kheiri, Mohammadreza Jannesari Ladani, Mohammadreza Piri Ardakani

**Affiliations:** Isfahan University of Medical Sciences, Isfahan, Iran

**Keywords:** Paediatric research, Musculoskeletal system, Medical research

## Abstract

Clubfoot is one of the common orthopaedic deformities. However, regardless of its’ treatment high success rate, recurrence of the deformity is a serious issue. The aim of this study is to evaluate if radiographic angles can be used for clubfoot recurrence prediction. This is a prospective study on 91 patients (134 feet) with mean age of 9.5 ± 2.3 days and male/female ratio of 2/1 on patients with congenital clubfoot admitted to our hospital. Pre and one-year post-tenotomy tibiocalcaneal (TIC-L), talocalcaneal (TC-L) and calcaneal-first metatarsal angles (C1M-L) in the lateral view of the patients' radiographs, and their recurrence status until three years were measured. Ten feet experienced relapse. The mean pre and one-year follow-up measurements of TC-L, C1M-L, and TIC-L angles were significantly different between patients who experienced relapse and others (*P* < .05). The cut-off points of 1.75 and 6.5 for one-year follow-up Pirani and Dimeglio scores for recurrence prediction were suggested respectively. Also, cut-off points of 26.5 and 79.5 for one-year follow-up TC-L and TIC-L angles for recurrence prediction were calculated, respectively. We demonstrated that the pre-tenotomy and one-year follow-up TIC-L, TC-L, and C1M-L angles are helpful in clubfoot recurrence prediction after Ponseti treatment.

## Introduction

Clubfoot is one of the common paediatric foot deformities. Ponseti method is known as an effective treatment method for idiopathic clubfoot^1–3^. The final goal of clubfoot treatment is to achieve a functional and plantigrade foot; However, factors affecting the deformity recurrence after the treatment have not been fully known, and treating its’ relapsed form is complex^4,5^. Most of the studies on clubfoot recurrence are focused on the subjective clinical severity scorings, however having simple and objective modalities such as radiographic angles might also be helpful. Whether radiographs should be part of the routine clubfoot treatment is still controversial^3,6^.

Brace-wearing is considered to be a crucial part of the Ponseti method^4,7^, and the foot’s abnormal anatomy, could lead to brace non-adherence (making it hard for the foot to fit within the brace) and further recurrences^8^. Therefore, it is important to recognize abnormal foot’s anatomy during follow-up sessions, and radiographs can be used for this purpose. Ponseti did not recommend using plain radiographs to evaluate treatment outcomes^9^; however, there are recent reports on the accuracy of radiographs for clubfoot follow-ups^3,8,10^. Zimmerman et al. reported that reproducible lateral radiographic angles measured by readers with different levels of experiences are reliable and they suggested these angles be used for recurrence risk prediction in patients with clubfoot^8^.

Our working hypothesis is to evaluate whether pre-tenotomy, and one-year follow-up radiographic angles in the lateral view can predict deformity recurrence in patients with clubfoot or not.

## Results

Of ninety-one patients (134 feet; 43 bilateral), there were 30 females, and 61 males. The mean age at initial treatment was 9.5 ± 2.3 days, and ten feet experienced recurrence of the deformity during the follow-up period of the study.

Cronbach’s alpha reliability analysis demonstrated that the reported measurements of talocalcaneal angle in the lateral view (TC-L) (α = 0.74), tibiocalcaneal angle in the lateral view (TIC-L) (α = 0.62), and calcaneal-first metatarsal angle in the lateral view (C1M-L) (α = 0.65) were reliable.

Using independent t-test, it was shown that the difference between mean pre-tenotomy TC-L, and TIC-L, and C1M-L angles, and their one-year follow-up measurements were significant (*P* < 0.001). The mean pre-tenotomy (*P*: 0.005), and one-year follow-up (*P*: 0.001) TC-L, and also mean pre-tenotomy (*P* < 0.001), and one-year follow-up (*P*: 0.02) C1M-L angles were significantly smaller in patients with relapse than others. Nevertheless, the mean pre-tenotomy (*P*: 0.001) and one-year follow-up (*P* < 0.001) TIC-L angles in patients with relapse were significantly greater than those without relapse (Table 1).

**Table 1:** The mean of pre and one-year post-tenotomy angles in patients with and without relapse (mean ± SD).

Angle	Relapse	No relapse	*P*-value
Pre-tenotomy Talocalcaneal angle in the Lateral view (TC-L)	7 ± 2.9	10.3 ± 3.5	0.005
One-year follow-up TC-L	21.1 ± 6.7	30.6 ± 4.7	0.001
Pre-tenotomy Tibiocalcaneal angle in the Lateral view (TIC-L)	101.1 ± 3.6	95.3 ± 5.1	0.001
One-year follow-up TIC-L	83.1 ± 6.1	72.1 ± 5.3	< 0.001
Pre-tenotomy Calcaneal-first metatarsal angle in the Lateral view (C1M-L)	3.9 ± 1.1	5.6 ± 1.2	< 0.001
One-year follow-up C1M-L	7.2 ± 2.8	9.8 ± 1.7	0.02

On the other hand, using independent t-test, it was demonstrated that the initial Pirani and Dimeglio scores and also the final (one-year follow-up) Pirani and Dimeglio scores were significantly different between patients who experienced recurrence of the deformity and others (*P* < 0.05) (Table 2).

**Table 2:** Mean initial and final Pirani and Dimeglio scores based on the recurrence status.

Variable (Mean ± SD)	Recurrence	No recurrence	*P*-value
Initial Pirani score	5.9 ± 0.2	5.4 ± 0.5	.004
Final Pirani score	1.7 ± 0.6	1.1 ± 0.4	.01
Initial Dimeglio score	18.4 ± 1.3	16.9 ± 1.5	.002
Final Dimeglio score	6.6 ± 0.7	5.9 ± 0.7	.004

Using Pearson correlation coefficient test, it was demonstrated that the Pirani score changes were not significantly related to any of the radiographic angles we measured; however, Dimeglio score changes were positively correlated with initial and one-year follow-up TIC-L angle (*P* < 0.001), and negatively correlated with initial and one-year follow-up TC-L and C1M_L angles (*P* < 0.05) (Table 3).

**Table 3:** Pearson correlation coefficient testing between Pirani and Dimeglio score changes and the measured angles.

Angle	Pirani score changes		Dimeglio score changes	
	r	*P*	r	*P*
Initial Talocalcaneal angle in the Lateral view (TC-L)	0.156	0.07	− 0.319	< 0.001
Follow-up TC-L	0.018	0.84	− 0.419	< 0.001
Initial Tibiocalcaneal angle in the Lateral view (TIC-L)	0.079	0.36	0.448	< 0.001
Follow-up TIC-L	− 0.056	0.52	0.368	< 0.001
Initial Calcaneal-first metatarsal angle in the Lateral view (C1M)	− 0.021	0.81	− 0.275	0.001
Follow-up C1M	− 0.124	0.15	− 0.244	0.004

Binary logistic regression was used for evaluating the simultaneous effects of all of our predicting factors. The result of Omnibus Tests of Model Coefficients was significant (*P* < 0.001), meaning that our full model is a significant improvement over the null model (the model in which we assume that none of the potential AVN predicting variables are significantly related to AVN occurrence). Also, Nagelkerke R Square value was 0.669, meaning that 66.9% of the variance in the dependent variable (AVN occurrence) could be explained by the independent variables (potential AVN predicting factors); In addition, the result of Hosmer and Lemeshow test was not significant (*P*: 0.179); however, in the regression model, none of the independent variables were significantly related to AVN incidence (Table 4).

**Table 4:** The results of binary logistic regression for evaluating the simultaneous effects of all of the potential AVN predicting factors.

Variables	*P*	Odds ratio
Pre-tenotomy Talocalcaneal angle in the Lateral view (TC-L)	.630	1.164
One year post-tenotomy TC-L	.150	.692
Pre-tenotomy Tibiocalcaneal angle in the Lateral view (TIC-L)	.411	1.175
One year post-tenotomy TIC-L	.512	1.111
Pre-tenotomy Calcaneal-first metatarsal angle in the Lateral view (C1M-L)	.182	.342
One year post-tenotomy C1M-L	.719	1.185
Initial Pirani score	.135	332.325
Final Pirani score	.511	.729
Initial Dimeglio score	.519	.575
Final Dimeglio score	.805	.667
Number of casts	.676	.667
Age	.251	.685
Sex	.902	.843
Unilateral_Bilateral	.901	.806

**Figure 1:**
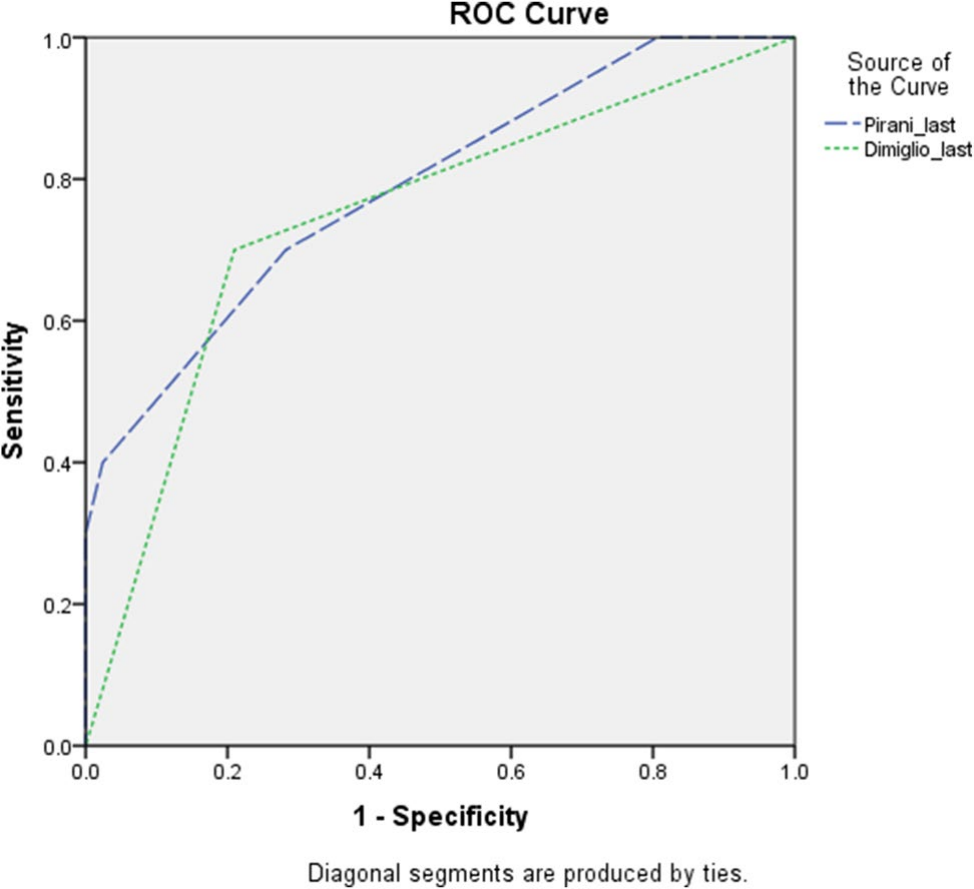
ROC curve for predicting recurrence based on the final Pirani score (blue curve), and the final Dimeglio score (green curve).

ROC curve analysis was used to calculate the cut-off points of final Pirani and Dimeglio scores based on the recurrence statues (Fig. 1). According to these analysis, the cut-off point of 1.75 for the final Pirani score with 70% sensitivity and 71.8% of specificity was reported. In other words, with this cut-off point, 70% of the patients with recurrence and 71.8% of the patients without recurrence could be accurately predicted.

Also the cut-off point of 6.5 for the final Dimeglio score with 70% sensitivity and 79% specificity was calculated (Fig. 1); in other words, with this cut-off point, 70% of the patients who underwent recurrence of the deformity and 79% of the patients who did not underwent any signs of relapse were accurately predicted.

**Figure 2:**
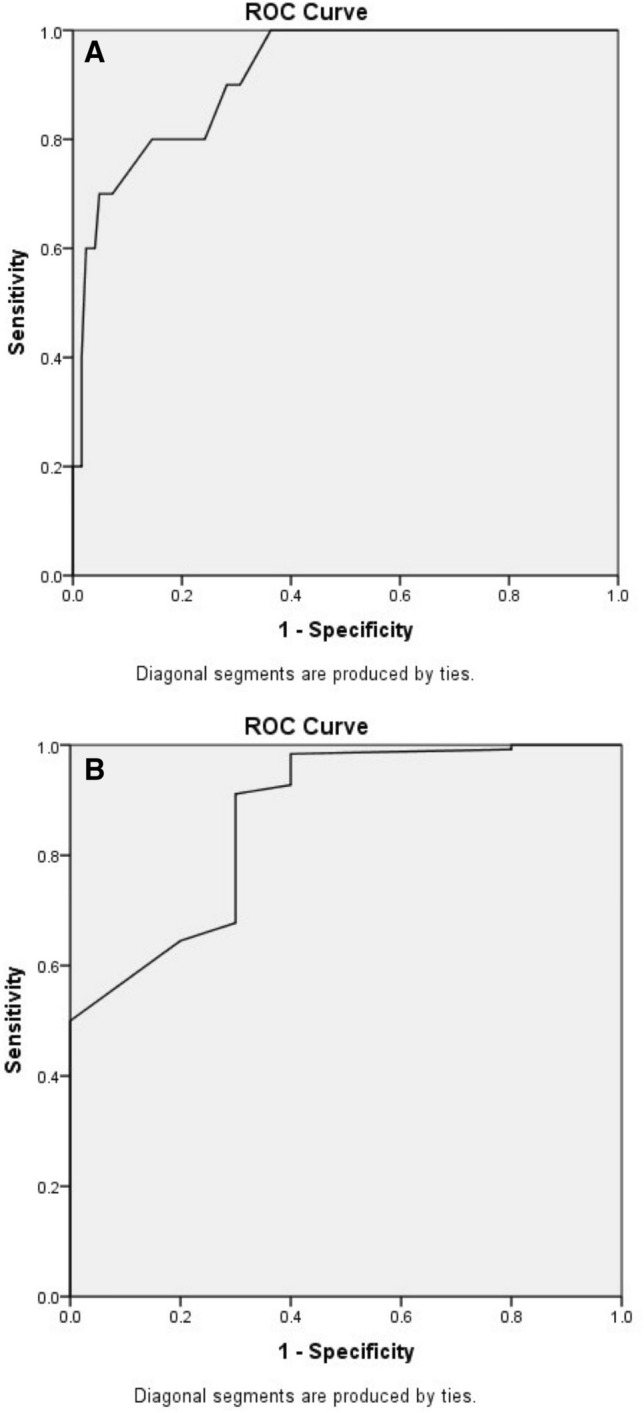
ROC curves for predicting recurrence based on the one-year follow-up talocalcaneal (upper curve) and tibiocalcaneal (lower curve) angles in lateral view.

In addition, the cut-off point of 26.5 with 82.3% sensitivity and 70% specificity was calculated using ROC curve for the recurrence chance prediction based on one-year follow-up TC-L angle (Fig. 2, upper ROC curve). Also, the cut-off point of 79.5 with 80% sensitivity and 85.5% specificity was calculated using ROC curve analysis for the recurrence chance prediction based on one-year follow-up TIC-L angle (Fig. 2, lower ROC curve).

## Discussion

The rate of clubfoot recurrence after Ponseti method is high, and factors causing it are still debatable^11^. Due to the high rate of recurrence, an objective evaluation of the feet via radiographs could help with recognising the early signs of recurrence, and having proper intervention in the right time. In this study, our hypothesis was confirmed, and we depicted that pre-tenotomy and one-year follow-up radiographic angles in the lateral view can be used for recurrence prediction, this challenges the Ponseti method, especially considering that Ponseti, himself, did not recommend the use of radiographs in his method; As mentioned, in our study, the pre-tenotomy and one-year follow-up TIC-L, TC-L and C1M-L angles were significantly different between the relapsed group and the non-relapsed group.

In addition, even though the binary regression model suggested no significant relationship between the potential risk factors and recurrence of the deformity, we believe that this result was obtained due to our relatively small sample size. Despite the fact that we used valid sample size calculations to estimate the number of feet needed for evaluating the effect of radiographic angles on clubfoot rccurrence separately, we believe that our sample size was not large enough to evaluate the simultaneous effects of all of the potential risk factors on deformity recurrence; however, our results still suggested correlations between the Dimeglio score changes and initial and one-year post-tenotomy TC-L, TIC-L and C1M-L angles. And considering the fact that Dimeglio score has been introduced as a valid predictor of recurrence of the deformity in many of the previous studies, its’ correlation with our angle measurements is another positive point for the confirmation of our hypothesis. Therefore, we suggest that at least two lateral radiographs (one before tenotomy, and one at the one-year follow up session), be added to the original Ponseti method, and TIC-L, TC-L, and C1M-L be measured accordingly.

Prasad et al. evaluated 12 different radiographic angles in anteroposterior and lateral view radiographs of 30 patients (50 clubfeet) who were between four to 16 years old, and were treated with posteromedial soft-tissue release. In their study, talocalcaneal angle (anteroposterior and lateral), talo-first metatarsal angle (anteroposterior and lateral), calcaneal-fifth metatarsal angle (anteroposterior), TIC-L, tibiotalar angle (lateral), talar dome flattening and talocalcaneal index were significantly correlated with the clinical scoring^12^. Our study differs from Prasad et al.’s study in the sense that we evaluated patients who were younger than two weeks old, and were treated with the Ponseti method. Therefore, due to the ossification of navicular later in life, measurement of the angles involving the navicular bone was not possible in our study. Also, we tried not to include angles involving the talar bone as it has been reported that in patients with severe clubfoot deformities, talar distortions could lead to angle measurement errors^13^ (except for the talocalcaneal angle which has been reported to be a good representative for the hindfoot alignment in many prior reports).

Kang et al. studied the radiological prognostic factors for clubfoot recurrence in 43 patients (64 feet) who underwent further selective soft tissue release (SSTR) due to their residual deformity or the recurrence of the deformity after initial Ponseti treatment. During 50.3± 28.5 months of follow up, preoperative talocalcaneal angle (anteroposterior and lateral), postoperative talo-first metatarsal angle (anteroposterior) and TC-L and TIC-L were associated with further recurrence after SSTR^14^. Their study inspired us to investigate the effect of almost the same radiologic angles on clubfoot recurrence in younger patients undergoing the Ponseti treatment as their first treatment approach.

TC-L represents hindfoot alignment, and it decreases in case of equinus or varus malalignment. It is recommended to measure the intrinsic contracture of the hindfoot with this angle^15,16^. Also, due to the possible measurement bias because of talar shape distortions in patients with severe deformities, calcaneus is preferred in the literature to talus in this regard (talo-first metatarsal angle vs. calcaneal-first metatarsal angel)^13^; therefore, we chose C1M-L angle as the representative of midfoot flexibility and cavus deformity in our study, which is a predictor for better brace compliance^17^. Midfoot and hindfoot parameters are reported to be of most importance for clubfoot recurrence prediction^15^. Residual equinus before and after tenotomy represented by TIC-L (ankle dorsiflexion) has also been reported to be an important predictive factor for further surgeries, and it can remain abnormal even years after the treatment^18^.

Percutaneous Achilles Tenotomy (PAT) is a crucial part of Ponseti method, and it is suggested that PAT be performed on all of the patients^19^. It lengthens the Achiles tendon which helps with correcting the residual equinus and reducing treatment duration, risk of recurrence, talar flattening or convex foot, and the number of surgical release required^20^. PAT was also recently added to the functional method, which is another effective treatment method for clubfoot. Nguyen khac et al. reported that the threshold of 75 degrees for the TIC-L can be used for determining which feet need PAT in the functional method^21^; Marleix et al. reported improvements in equinus and talocalcaneal divergence after PAT, using sonographic imaging^19^.

Zimmerman et al. reported that lateral angle of dorsiflexion, TIC-L, TC-L and lateral talo-firstmetatarsal (which measures the alignment of the forefoot with respect to the hind-foot and determines the presence of a mid-foot breach or rocker-bottom deformity) angles can be reliably measured in patients with clubfoot and in their study, these angles demonstrated significant changes before and after PAT^8^. Li et al. reported that ankle dorsiflexion and TC-L both increased after PAT which in their study, lead to improved subtalar joint and alignment^22^. Their study was unique in terms of being the first study on TC-L changes after PAT. We only measured the pre-tenotomy and one-year follow (not the immediate post-tenotomy) angles as the ethics committee allowed us to add only two further radiographs to the original Ponseti method. Our results confirm the general concept of Li et al.’s study, and also suggest that one-year follow-up radiographs are useful in recurrence prediction.

Same as our results, Mishima et al. suggested that pre-tenotomy TC-L, and TIC-L, and C1M-L angles in maximum dorsiflexion, had the most positive predicting value for rigid relapse^15^. Richards et al. studied TIC-L and TC-L angles of 312 clubfeet with different initial severities undergoing Ponseti or French methods at 18–24 months old. Based on their results, these angles were not valuable for relapse prediction^23^. In our study, we treated all of the patients based on the Ponseti protocol. In addition, we only included patients with severe or very severe initial deformities, as the initial severity of the deformity is shown to be related to recurrence of clubfoot^24–26^.

In a retrospective study, O’Holloran et al. reported that increased dorsiflexion could predict better midfoot flexibility and probably better brace compliance. In addition, 15 degrees was introduced as the cut-off point for foot dorsiflexion for recurrence prediction^17^. Our study confirmed their results by using the TIC-L angle as the representative of ankle dorsiflexion; however, we prospectively studied a larger number of patients with only severe and very severe deformities, which adds more credibility to our work.

Li.et al. reported that talocalcaneal angle was reduced in both anteroposterior and lateral views in the relapsed group in their study. However, after applying logistic regression, they reported that neither of the mentioned angles had predictive values in recurrence prediction, and only talo-first metatarsal angle in anteropsterior view, and TIC-L angle had positive predictive values for relapse. This was a well-evaluated study on radiographic angles in patients with clubfoot, and they recommended further study on the subject be done in the future^6^. We solely measured the angles in the lateral view because our ethics committee allowed us to perform only two further radiographs on the patients, and as there were reports on the lack of anteroposterior talocalcaneal angle congruency with the real anatomical relationship between talus and calcaneus demonstrated by 3D CT scan, we decided to apply only lateral radiographs for the patients^27^; even though, we compared the angles only in the lateral view, our results confirmed Le et al.’s study. In addition, we reported cut-off points for one-year follow-up TC-L and TIC-L angles based on recurrence prediction. We demonstrated that, the cut-off point of 26.5 degrees for one-year follow-up TC-L angle could accurately predict 82.3% of the recurrences, and 70% of non-recurrences. Also, with the cut-off point of 79.5 for one-year follow-up TIC-L angle, 80% of recurrences, and 85.5% of non-recurrences could accurately be predicted.

Fridman et al. studied 50 patients (71 clubfeet) who were between six and 26 months old, and were treated by soft tissue release. They reported that during an average follow up of 77.03 months, only talo-first metatarsal angle and calcaneal-second and fifth metatarsal angles in the anteroposterior view were related to the feet functional outcomes. They suggested that these angles be considered for introduction of a new functional system for patients with clubfoot^13^. With all due respect to their study, we believe that our study provides a more reliable methodology as we only entered patients who were less than two weeks old, and had only severe and very severe initial deformities. Also, as mentioned, and reported by Fridman et al., functional scoring systems change and develop over time, and therefore, we believe that it is more reliable to evaluate the treatment outcomes based on one unchangeable criteria (for example recurrence of the deformity as we used in our study) than to introduce new functional systems.

Last but not least, our results suggested new cut-off points for the one-year post-tenotomy Pirani score and Dimeglio scores for recurrence prediction. Based on our results with the cut-off point of 1.75 for the final Pirani score, 70% of the patients with recurrence and 71.8% of the patients without recurrence could be accurately predicted. Also with the cut-off point of 6.5 for the one-year follow-up Dimeglio score, 70% of the patients who experienced recurrence of the deformity and 79% of the patients who did not underwent any signs of relapse were accurately predicted. These results can be used alongside the suggested cut-off points of radiographic angles for recognizing the patients who are at higher risk for recurrence, in order to follow-up with them more strictly.

This study has some limitations. Our study has an undeniable selection bias, since our hospital is the only medical centre in the city with trained professionals in treating severe clubfoot, mostly advanced cases are admitted to our hospital. Also, even though, we relied on the reports of the most expert person in our team, we believe that double checking the measurements by another professional could have decreased the probable measurement bias which we may not have encountered. In addition, the cases were followed up for three years, and further recurrences after this age were not recorded. Also, we relied on the parents’ reports for the brace compliance, as we did not have sensors measuring the brace wearing time during the study, this however, appears to be the limitation in many of other published studies. Further research on this subject with larger sample sizes, longer follow ups and using sensors within the casts is recommended.

## Conclusion

Pre-tenotomy and one-year follow-up TIC-L, TC-L, and C1M-L angles, could be used for recurrence prediction in patients with severe, and very severe clubfoot deformities. We suggest that radiographs be added to the pre-tenotomy, and one year follow up stages of the Ponseti method. In addition, we recommend that feet with one-year follow-up TC-L angle of 26.5, and smaller degrees and one-year follow-up TIC-L angle of 79.5, and greater degrees be followed up more strictly.

## Patients and methods

This study was approved by the institutional Review Board of Isfahan University of Medical Sciences in Iran (IRB code: IR.MUI.MED.REC.1397.274). Also it was assured that all methods used in this research were performed in accordance with the declaration of Helsinki. Sample size was calculated to be at least 82 feet using the following formula:

$$n = \frac{{\left( {{\text{Z}}1 + {\text{Z}}2} \right)^{2} *(1 - r^{2} )}}{{r^{2} }} + 2$$

In this formula, Z_1_ is the constant set by convention according to accepted α error with 95% confidence interval (it is 1.96 in our study). Z_2_ is the constant set by convention according to power of the study. As the power of our study is 80%, Z_2_ would be 0.84. And $r$ is the expected correlation coefficient between the radiographic angles and the Pirani and the Dimiglio scores, its’ absolute value in our study is 0.30.

In this study, the data set of 91 patients with clubfoot was collected at Kashani hospital in Iran from January 2014 to December 2017. Exclusion criteria were: lack of consent from patients’ parents for participating in the study, having older than fifteen days of age, receiving treatment in other medical centres, not having severe or very severe initial deformity based on the Dimeglio scoring system.

Patients were treated based on the protocol proposed by Ponseti et al.^28^. The method consists of serial manipulation and casting with seven days’ interval until achieving proper correction in midfoot cavus, forefoot adduction, and hindfoot varus. All of the patients in our study underwent PAT after their initial deformity correction. Then long leg cast was applied for all patients for an extra three weeks and then Denis-Brown braces was administered for them to be worn 23-hours a day for the first three months, and then 20-hours a day for the second three months, and 16-hours a day for the third three months, then 12-hours a day for the last three months of treatment, and then reverse last shoes were administered to be worn in daytime and Denise Brown brace was advised to be worn during night time until patients reached four years of age.

**Figure 3:**
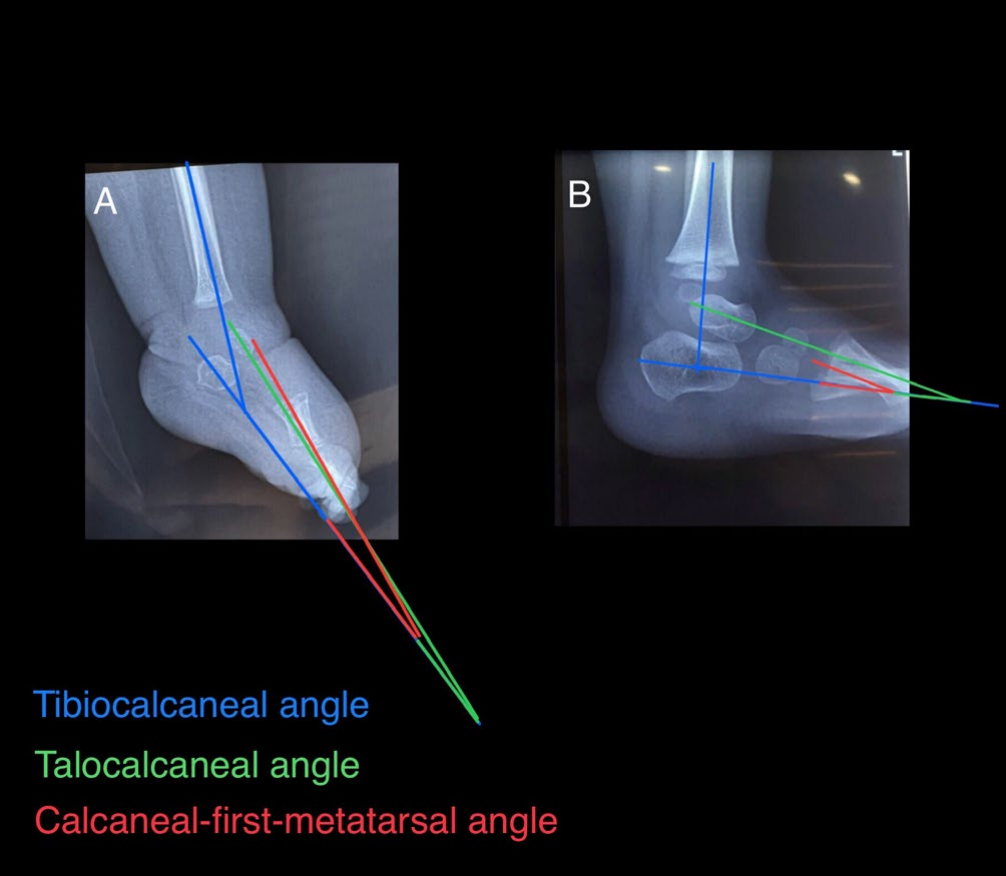
Angle measurements depicted on pre-tenotpmy (**A**), and one-year post-tenotomy (**B**) radiographs

TC-L, TIC-L, and C1M-L radiographic angles before and one-year after PAT with ankle in maximal dorsiflexion were measured (Fig. 3). Patients' demographic information as well as their initial and one-year post-tenotomy follow-up Pirani and Dimeglio scores and initial and one-year follow-up radiographic angles were measured and reported personally by one expert attending paediatric orthopaedic surgeon. In addition, all of the patients were followed up by him for three years after the last cast removal.

In this study,* P* < 0.05 was used as the significance level, and SPSS version 22, Chicago, was used for statistical analysis. It is noteworthy to mention that in our statistical analysis, patients with bilateral clubfoot were counted twice as the incidence of clubfoot is iatrogenic and the occurrence of clubfoot in one side is independent of the occurrence of the deformity in the other side.

### Ethical approval

Ethical approval was obtained from the research ethics committee of Isfahan University of Medical Sciences. Written informed consent to participate was obtained from children’s’ parents before the start of the research.

## Data Availability

The data that support the findings of this study are available from Isfahan University of Medical Sciences but restrictions apply to the availability of these data, which were used under license for the current study, and so are not publicly available. Data are however available from the authors upon reasonable request and with permission of Isfahan University of Medical Sciences.
